# Cross-Resistance: A Consequence of Bi-partite Host-Parasite Coevolution

**DOI:** 10.3390/insects9010028

**Published:** 2018-02-26

**Authors:** Tilottama Biswas, Gerrit Joop, Charlotte Rafaluk-Mohr

**Affiliations:** 1 Institute of Insect Biotechnology, Justus-Liebig University Giessen, Heinrich Buff Ring 29-32, 35392 Giessen, Germany; g.joop@gmx.de; 2 Institute of Animal Ecology, Leuphana University of Lüneburg, 21335 Lüneburg, Germany; 3 Department of Zoology, University of Oxford, South Parks Road, Oxford OX1 3PS, UK

**Keywords:** route of infection, RT-qPCR, *Tribolium castaneum*, *Beauveria bassiana*, *Bacillus thuringiensis*, *Pseudomonas entomophila*, multiple parasites

## Abstract

Host-parasite coevolution can influence interactions of the host and parasite with the wider ecological community. One way that this may manifest is in cross-resistance towards other parasites, which has been observed to occur in some host-parasite evolution experiments. In this paper, we test for cross-resistance towards *Bacillus thuringiensis* and *Pseudomonas* *entomophila* in the red flour beetle *Tribolium castaneum*, which was previously allowed to coevolve with the generalist entomopathogenic fungus *Beauveria bassiana*. We combine survival and gene expression assays upon infection to test for cross-resistance and underlying mechanisms. We show that larvae of *T. castaneum* that evolved with *B. bassiana* under coevolutionary conditions were positively cross-resistant to the bacterium *B. thuringiensis,* but not *P. entomophila*. Positive cross-resistance was mirrored at the gene expression level with markers that were representative of the oral route of infection being upregulated upon *B. bassiana* exposure. We find that positive cross-resistance towards *B. thuringiensis* evolved in *T. castaneum* as a consequence of its coevolutionary interactions with *B. bassiana*. This cross-resistance appears to be a consequence of resistance to oral toxicity. The fact that coevolution with *B. bassiana* results in resistance to *B. thuringiensis,* but not *P. entomophila* implies that *B. thuringiensis* and *B. bassiana* may share mechanisms of infection or toxicity not shared by *P. entomophila*. This supports previous suggestions that *B. bassiana* may possess Cry-like toxins, similar to those found in *B. thuringiensis*, which allow it to infect orally.

## 1. Introduction

In nature, hosts are likely to exist within a complex community and interact with multiple parasites [1,2,3]. Close bipartite species interactions established over evolutionary time are likely to impact other ecological interactions within the community [4,5,6,7], including those with other parasite species [8]. In spite of this, to date, few experimental studies investigate the consequence of bipartite host-parasite interactions on host interactions with other parasites [9,10,11,12,13]. Many host-parasite coevolution experiments attempt to understand the dynamics [14,15], adaptations [16,17,18], and underlying genetic mechanisms [16,19,20,21] of host responses in the context of the parasite it coevolved with. Such evolutionary interactions can impact host traits important in the wider context of its environment [22]. For example, larvae of *Drosophila melanogaster* evolved with the parasitoid *Asobara tabida* displayed reduced competitive ability under high competition [22]. However, broad-scale effects of coevolution on hosts’ interactions with a wider community of parasites as a consequence of host-parasite coevolution are yet to be investigated. Cross-resistance is one such broad-scale effect [23].

Cross-resistance is a host defence mechanism that develops as a response to parasite infection, whereby host evolutionary interactions with one parasite (A) leads to either host resistance, tolerance, or hyper-susceptibility to another parasite (B or C) [23]. When evolutionary interaction with a parasite (A) results in the host being resistant to previously un-encountered parasites (B) it is termed as positive cross-resistance [23]. For example, Martins et al. [10] reported that experimental evolution of the fruit fly *Drosophila melanogaster* with the bacterium *Pseudomonas entomophila* (A) results in the flies being resistant to *P. putida* (B). Alternatively, since mounting an immune response and developing resistance can come with associated life-history costs [24,25], evolutionary interaction with a parasite may result in the host being hyper-susceptible to a new and previously un-encountered parasite (C), leading to negative cross-resistance [23]. Martins et al., [10] also observed that the evolved flies were more susceptible to infection by viruses (flock house virus (FHV) and *Drosophila* C virus (DCV)) as compared to their control counterparts. It was proposed that the higher survival of flies evolved to *P. entomophila* upon infection with *P. putida* comes at a cost which is manifested in the form of hyper-susceptibility to viral infections (C) [10]. In another study where *D. melanogaster* was allowed to adapt to DCV for 20 generations, a genome-wide analysis of the evolved flies revealed that their cross-resistance to the cricket paralysis virus (CrPV) and the FHV are mediated by the gene *Pastrel* and two other loci, namely Ubc-E2H and CG8492 [13]. The authors further confirmed this observation by knocking out these candidates in flies using RNAi, which resulted in significantly higher mortality when exposed to the aforementioned viruses [13]. However, mechanisms of cross-resistance are poorly understood and rarely studied in other host-parasite systems. Host evolution with endosymbiontic bacteria can also influence interactions with potential parasites. These live within the body of the insect host and have also been observed to confer protective benefits against parasites. The vertically transmitted falcultative endosymbiont *Regiella insecticola* increases host resistant to fungal parasites *Pandora aphididis* [26] and *Zoophthora occidentalis* [27] in the pea aphid *Acryrthosiphon pisum*. The male-killing endosymbionts of *D. melanogaster*, Spiroplasma, and Wolbachia, protect flies by reducing the survival of parasitoid wasps [28].

Cross-resistance can occur at the evolutionary (manifested across generation) or ecological (manifested within generation) level [23]. At the evolutionary level, it has been proposed that cross-resistance to one parasite (A) is connected with resistance to a different parasite (B, C), by means of shared defence mechanism [13]. This is shown by *D. melanogaster* evolved to DCV when exposed to CrPV and FHV, as discussed before [13]. At the ecological level, cross-resistance is the result of the activation of immune defence caused by the previous exposure to a different parasite (A) [23]. For instance, within the same generation, prior exposure of the mosquito *Anopheles gambiae* to the microsporidian parasite *Vavria culicis* (A) results in the mosquitoes being more resistant to *Plasmodium berghei* (B)*,* when compared to control mosquitoes, due to an enhanced melanisation response [1]. Within the context of this paper we refer to the evolutionary definition of cross-resistance.

To date, only a handful of studies have tested whether, under simple experimental evolution conditions, host resistance leads to general cross-resistance effects upon exposure to different parasites [9,11,23,29]. Fellowes et al. [9] showed that populations of *D. melanogaster* experimentally evolved with the parasitoid wasp *Leptopilina boulardi* demonstrated positive cross-resistance towards *L. heterotoma,* but no difference in resistance towards *A. tabida*. Kraaijeveld et al. [23], reported that *D. melanogaster* experimentally evolved to *A. tabida* exhibited no change in resistance towards either the fungus *Beauveria bassiana* or microsporidian *Tubulinosema kingi*. Bentz, and colleagues [29] reported no difference in *D. melanogaster*’s resistance towards Drosophila sigma virus after experimental evolution with *Bacillus cereus*. Similarly, the greater wax moth *Galleria mellonela* evolved with the fungus *Beauveria bassiana*, displayed no difference in resistance to the fungus *Metarhizium anisopliae* [11]. It remains unclear why hosts are cross-resistant to some parasites and not others.

The few studies reporting cross-resistance have tried to understand the underlying mechanisms at play, such as specificity of route of infection [10] or the genetic basis of resistance [13]. In cases that have reported positive cross-resistance, the first (A) and the subsequent parasite (B) the host was exposed to were closely related [9,10,13], resulting in the host employing similar immune mechanism against both. Furthermore, in studies that have reported negative cross-resistance or no difference in resistance, the first (A) and the subsequent parasite (B,C) to which the host was exposed to, belonged to different taxonomic groups [10,13,23,29]. These observations hint that relatedness of the parasite is of relevance for the occurrence of different types of cross-resistance. In *D. melanogaster* that evolved with *P. entomophila* by oral infection, Martins et al. [10] observed that positive cross-resistance to the closely related *P. putida* was observed only upon oral infection and not when the flies were infected systemically (i.e. cuticular breaching); indicating that route of infection might be an important factor in cross-resistance. Adaptations to different routes have been shown to have different genetic underpinnings. In a study by Behrens et al. [30], it was shown that *Tribolium castaneum* has different gene expression profiles upon oral and systemic infection by the same parasite.

Although evolutionary studies of cross-resistance have been carried out, cross-resistance in a host arising out of experimental host-parasite coevolution (i.e., both the host and parasite are allowed to adapt to each other over time [31]) has so far not been investigated. Here, we investigate cross-resistance in hosts adapted to a single parasite species (A) under coevolutionary conditions upon exposure to unrelated parasites (B, C). We conducted the present study with the following aims: (i) Does coevolution with a parasite result in cross-resistance to an unrelated parasite? (ii) Does the route of infection play a role in cross-resistance to unrelated parasites? The beetle *T. castaneum,* which had evolved under conditions allowing for host-parasite coevolution with the fungal parasite *B. bassiana* [32,33] was the host for the experiments mentioned in our paper. During the evolution experiment [32,33], *B. bassiana* was present in the environment of the beetles, thereby, allowing for infection to occur naturally. We performed survival assays with the entomopathogenic bacteria *Bacillus thuringiensis* bv. *tenebrionis* (Gram positive; *B. thuringiensis* henceforth) and *P. entomophila* (Gram negative), and additionally with non-evolved *B. bassiana* and tested the gene expression profiles of *T. castaneum* evolved with *B. bassiana* upon exposure to *B. thuringiensis*, *P. entomophila* and non-evolved *B. bassiana*. *B. thuringiensis* and *P. entomophila* are phylogenetically distinct entomopathogenic bacteria, belonging to distinct clades of the bacterial phylogenetic tree [34]. Our results indicate that cross-resistance evolved towards *B. thuringiensis* as a consequence of coevolution with *B. bassiana* and that cross resistance is potentially due to a shared route of infection between *B. bassiana* and *B. thuringiensis*.

## 2. Materials and Methods

### 2.1. Host

**Figure 1:**
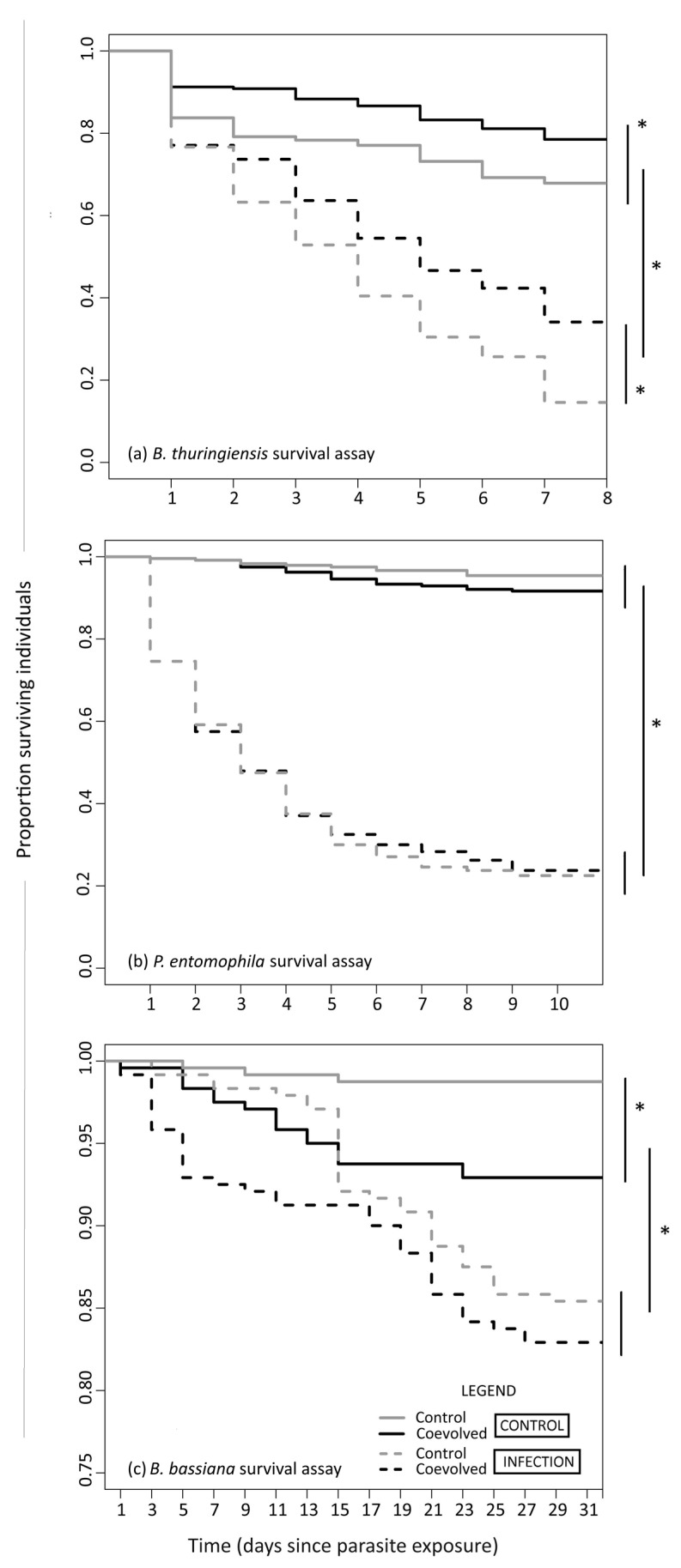
Survival of *B. bassiana* coevolved beetles upon infection upon exposure to non-evolved (**a**) *B. thuringiensis*, (**b**) *P. entomophila,* and (**c**) *B. bassiana.* * denotes significance between groups. Note: y-axis in (**a**) starts from 0.75 for better visualization of data.

*The red flour beetle T. castaneum* (from ancestral CRO1 population [35]) were used as hosts. Experimental coevolution was performed prior to the start of the experiments described in this paper. *T. castaneum* hosts and the parasite *B. bassiana* were allowed to evolve in each other’s presence at a starting concentration of 10^8^ spores·g^−1^ of *B. bassiana* for 13 host generations [32]. *B. bassiana* initially had a strongly negative influence on host fitness [33]. Control treatments were run in parallel without *B. bassiana* in the environment. Seven independent selection lines were produced for each treatment. Coevolution was allowed to occur for 13 host generations, followed by one generation of relaxed selection without the presence of *B. bassiana* in any regime resulting in generation 13. After which F1 adults were generated and maintained without the presence of *B. bassiana*, in clean flour-mix [32] to minimise potential maternal effects and the influence of transgenerational immune priming [36]. A schematic of the evolution experiment can be found in [32], Figure 1. All of the survival assays and gene expression experiments here were conducted on the F2 of beetles from generation 13, maintained under standard rearing conditions for *T. castaneum* (dark, at 32 °C with 70% relative humidity) in a 5% *w*/*w* mixture of brewer’s yeast and organic wheat flour (type 405, Alnatura). The flour-mix serves as the beetles’ immediate environment, as well as food resource. Throughout this paper, we refer to the hosts as ‘Control’ for beetles originating from populations evolved without the presence of *B. bassiana* and ‘Coevolved’ for those originating from populations forced to evolve with *B. bassiana* in the environment. Both ‘Control’ and ‘Coevolved’ selection regimes were simultaneously performed on seven replicate populations.

### 2.2. Parasites

All of the parasites were cultivated from their respective glycerol stocks (50% glycerol, Carl-Roth) stored at −80 °C. *B. thuringiensis* was cultured strictly as per the protocol in Milutinovic et al. [35], allowing for the production of spores which in turn produce *Cry* toxins. *P. entomophila* was grown overnight in LB medium (Carl-Roth) in a 250 mL culture flask at 30 °C and under shaking conditions of 200 RPM. The overnight culture of *P. entomophila* was centrifuged at 3200 G-force to obtain bacterial pellets while the culture supernatant was discarded. The pellets thus obtained were suspended in Phosphate Saline Buffer (pH = 7) prior to use in survival assay. Non-evolved *B. bassiana* was plated on Potato-Dextrose agar (Carl-Roth) and stored at room temperature for 2–3 weeks prior to spore collection. 

### 2.3. Survival Assays

Of the seven replicates beetle populations per selection regime, five populations from the ‘Control’ and ‘Coevolved’ regimes were used for the survival assays. F1 adult beetles belonging to generation 13 of the coevolution experiment [32] were set up for mating and egg laying for three days. At the end of this period, adults were removed and the eggs were allowed to develop under standard beetle rearing conditions for 10 days. Throughout this paper, we refer to treatments in the infection and gene expression experiments as CONTROL (not exposed to parasites) and INFECTION (exposed to a specific number of parasites). Survival assays were performed on the resulting F2 larvae, to control for maternal effects, on the 10th day post adult removal. Forty larvae per beetle population per treatment ((5 × 40) + (5 × 40) = 400 in total) were used in each of the survival assays.

*B. thuringiensis* survival assay was performed in a 96-well plate (Greiner Bio-one) setup with a spore concentration of 5 × 10^9^ spores·mL^−1^ of flour-mix & Phosphate buffer saline (PBS, pH = 7) solution, in line with Milutinovic et al. [35]. On the first day, 96-well plates containing 40 µL per well of either flour-mix with *B. thuringiensis* spore suspension or flour-mix with sterile Phosphate Buffer solution (PBS; pH = 7.0) were prepared, which were dried overnight at 50 °C. The next day, one larva per well was placed in the dried 96-well plates, with each plate containing two replicate populations per treatment. Finally, each plate was sealed with transparent sticky tape and three holes using board pins were punctured on top of each well to allow air circulation. These were then put in plastic boxes that were stored under standard rearing conditions. Each 96-well plate that was set up contained only one treatment and larval survival was observed for seven days on a daily basis. 

For the *P. entomophila* survival assay, larvae were infected systemically the protocol by Roth et al. [37]. Individual larva was pricked in the pronotum, on the left dorsal side, with a needle (diameter = 0.05 mm) dipped in either 5 × 10^9^ spores·mL^−1^ of *P. entomophila* spore suspension or sterile PBS, for INFECTION and CONTROL treatments, respectively. Post pricking, larvae were individualised in 96-well plates containing 40 µL of flour-mix & PBS solution dried overnight at 50 °C in a manner similar to that for *B. thuringiensis* survival assay. Survival was recorded every day for a period of 10 days. 

For *B. bassiana* survival assay, larvae were placed individually in small glass vials (40 mm × 13 mm, Carl-Roth) with 0.17 g mixture of flour-mix containing 10^8^ of *B. bassiana* spores·g^−1^ for INFECTION treatment and vials were capped with cotton wool stoppers (Carl-Roth). For CONTROL, larvae were placed in glass vials containing just 0.17 g of flour-mix. Since *B. bassiana* is a slow killer of *T. castaneum*, survival was monitored every alternate day for a period of 30 days (See Supplementary Materials File 1 for daily survival for each assay).

### 2.4. Host Treatment Prior to Investigating Gene Expression

Since there was no difference between the different replicate populations within each treatment, we selected two replicate *B. bassiana* coevolved beetle populations that showed the highest numerical survival (absolute number of individuals surviving out of the 40 individuals treated per population) at the end of the assay, for investigating gene expression as logistically we could not test all populations. Adults were set up for mating and egg laying as mentioned before and the larvae could develop. Following this, larvae were infected as per the methods that are described in the survival assay with *B. thuringiensis*, *P. entomophila* and *B. bassiana* individually. For CONTROL, the coevolved larvae were not exposed to parasites but handled similarly. Post treatment, the larvae were placed under standard rearing conditions for beetles. For each parasite, larvae were sampled from both CONTROL and INFECTION treatments 12 and 24 h post exposure. These two time-points were chosen based on previous transcriptomic studies using *B. thuringiensis* [30] and *B. bassiana* [32]. After collection larvae were immediately snap frozen in liquid nitrogen and stored at −80 °C until RNA extraction.

### 2.5. RNA Extraction and cDNA Synthesis

RNA was extracted from pools of 15 larvae (per treatment and time-point). The samples were homogenized (2 × 30 s) in 2 mL Eppendorf tubes with one 5 mm diameter stainless steel bead (Qiagen, Hilden, Germany) per tube. Each tube contained 400 µL of TRI reagent (Sigma-Aldrich (Merck), Darmstadt, Germany), using Tissue–lyser II (Qiagen) at 30 Hz for 2 × 30 s. Further treatment for RNA extraction was performed on this homogenate using Direct-zol™ RNA MiniPrep kit (Zymo Research, Irvine, CA, USA) as per the specifications in the manufacturer’s protocol. RNA concentration and purity were determined spectrophotometrically (Take3, BioTek, Bad Friedrichshall, Germany) and quality was checked using agarose gel electrophoresis with 1× TAE buffer. RNA samples with a quality ratio of A_260_/A_280_ (residual phenolic contamination) and A_260_/A_230_ (nucleic acid purity) ~ 2 containing two sharp bands representing 23S and 18S rRNA were used for cDNA synthesis, in accordance with MIQE guidelines [38]. Unsatisfactory RNA samples were concentrated using 100% ethanol and 3 M Sodium Acetate (Thermo-Fisher Scientific, Schwerte, Germany), as per the standard protocol. For cDNA synthesis, 2 μg of total RNA, oligo (dT)_18_ primers and the reagents from the First Strand cDNA Synthesis Kit (Thermo-Fisher Scientific) were used in accordance with the manufacturer’s protocol. The resulting cDNA was diluted to a working concentration of 10 ng·μL^−1^, and stored in separate 1 mL Eppendorf tubes at −80 °C until used.

### 2.6. Candidate Genes and Primer Validation

We surveyed existing literature for *T. castaneum* genes expressed post infection and decided on representative candidate genes enlisted in Supplementary Materials File 2, Table S1. We selected candidate genes based on a survey of existing gene expression studies which span RT-qPCR [39], transcriptomic [30] and functional analysis [40,41,42] approaches. The genes tested represent stress (Hsp90, p450), phenoloxidase (PO) (Laccase-2 (Lac-2; [40]) and Apolipophorin-III (Apo-III; [30,42])) and antimicrobial peptides (Attacin-2 (Atta-2) & Defensin-3 (Def-3) [39]). Additionally candidates for external immune defence (quinone-related; Gt39 [43]), expressed upon fungal challenge (Thaumatin-like; Thaumatin [39]), for innate immunity (Lysozyme (Lyso-4; [30])) and chitin metabolism (chitin deacetylase (TcDA6; [41])) were analysed. Among these, markers for oral (Apo-III & ObpC-12) and systemic (Hsp-90 & p450) routes of infection were used to test our hypothesis that cross-resistance is route dependent. Gene-specific primers (biomers.net) were designed using Oligo Explorer v1.1.2 (available online at http://www.genelink.com/tools/gl-oe.asp). All primers were designed to have 19–23 nucleotides with a T_m_ ~60 °C and amplification products of length 70–150 base-pairs (bp). The volume of the reaction was 10 μL, containiing 5 μL Power SYBR Green PCR Master Mix (Applied Biosystems, Darmstadt, Germany), 1 μL (10 ng) of cDNA and specific forward and reverse primer different concentrations (150 nM, 300 nM, 450 nM and 900 nM in a full-factorial manner), with remaining volume scaled up with water in order to determine the optimum forward to reverse primer ratios.Primers were tested on cDNA prepared from stock CRO1 *T. castaneum* in a melt curve assay to determine the optimum forward to reverse primer ratio, as mentioned in Supplementary Materials File 2, Table S2, followed by a standard curve assay using the StepOnePlus Real-Time PCR System (Applied Biosystems) to determine primer efficiencies (Supplementary Materials File 2, Table S1). The total reaction volume was 10 we used cDNA concentrations spanning 0.001 to 100 ng in 5-fold dilutions with the cycling conditions as described in the following for performing standard curve analyses. Hot-start PCR with denaturation at 95 °C was run for 10 min followed by 40 cycles of extension at 95 °C for 15 s and at 60 °C for 60 s. Finally melt curve analysis was run with a step-wise temperature increment from 60 °C to 95 °C in steps of 0.5 °C. Primer efficiency was calculated using StepOne Software v2.3 (Thermo-Fisher Scientific) and only primers with an efficiency of 85–110%, regression fit of *R^2^* ≥ 0.98 and a single sharp melt curve peak corresponding to specific amplification were used for RT-qPCR experiments. Additionally, all primers were tested with water and stock CRO1 mRNA as template to check for primer dimers and unspecific amplifications, respectively (here *C_t_* or threshold cycle was set at *C_t_* ≥ 40 as per MIQE guidelines [38]). Only the primer pairs for the amplifications occurred at *C_t_* ≤ 20 were included in the analysis.

### 2.7. Gene Expression Using Quantitative PCR

Quantitative real-time RT-PCR was carried out on a StepOnePlus system (Applied Biosystems) using optical 96-well plates (Applied Biosystems) and covered with MicroAmp^TM^ optical adhesive film (Thermo Fischer Scientific). The total reaction volume of 10 μL contained 5 μL Power SYBR Green PCR Master Mix (Applied Biosystems), 1 μL (10 ng) of cDNA, specific forward and reverse primer concentrations (Supplementary Materials File 2, Table S2) with remaining volume scaled up with water. All of the reactions were carried out in three technical replicates under the reaction conditions stated above. Baseline correction was performed automatically by StepOne Software v2.3. Reactions for reference genes Rps3 and Rps18 [44] were performed on every 96-well plate setup, to normalise gene expressions. Additionally, two technical replicate reactions were performed in each of the 96-well plates, for no-reverse transcription and RNA control (to control for unspecific amplification with genomic DNA), in accordance with the MIQE guidelines [38]. Here, amplifications before ≤ 30 *C_t_* were included in the analysis.

### 2.8. Statistical Analysis

All data were statistically analysed using R software version 3.2.3 (Vienna, Austria) for statistical programming [45].

### 2.9. Survival Analysis

Since the survival data did not meet the assumptions of normality, we performed non-parametric Kaplan Meier analysis using the package ‘survival’ in R [46]. Multiple pairwise comparisons of survival curves were performed using an adaptation of a code by Terry Therneu, the results of which were corrected using Holm method.

### 2.10. Gene Expression Analysis

Gene expression data from RT-qPCR (Supplementary Materials Files 3 and 4) were analysed using the MCMC.qpcr R package [47,48], which implements a generalized linear mixed model analysis of qPCR data. We used the ‘classic’ mode, which normalizes the expression data of different candidate genes relative to ‘control’ genes (reference genes). We constructed a full factorial model with ‘Treatment’ and ‘Timepoint’ as interaction terms. P-values were adjusted for multiple comparisons using the Benjamini & Hochberg correction method implemented in the ‘p.adjust’ function d in R.

## 3. Results

### 3.1. B. bassiana Coevolved Beetles Are Positively Cross-Resistant to B. thuringiensis 

Survival of F2 larvae of generation 13 beetles from the coevolution experiment was recorded for a period of 7, 10, and 30 days post exposure to, *B. thuringiensis*, *P. entomophila*, and *B. bassiana*, respectively. 

In the *B. thuringiensis* assay, survival of groups within INFECTION and CONTROL treatments were significantly different from each other (χ^2^ = 224, 3 Degrees of Freedom (DF) = 3, *p* < 0.001). Pairwise comparison of Kaplan-Meier curves (Figure 1a and Table 1) show that ‘Control’ and ‘Coevolved’ groups differ in CONTROL treatment (*p* = 0.05), and highly differ in INFECTION treatment (*p* < 0.001). Here, higher survival of coevolved beetles upon INFECTION, in comparison to their control counterparts indicates positive cross-resistance to *B. thuringiensis* upon oral infection. 

In the *P. entomophila* assay, survival of groups in INFECTION and CONTROL treatments were significantly different from each other (χ^2^ = 544, DF = 3, *p* < 0.001). Pairwise comparison of Kaplan-Meier curves (Figure 1b and Table 1.) show that ‘Control’ and ‘Coevolved’ groups do not differ from each other in either CONTROL (*p* = 0.19) or INFECTION (*p* = 0.80). This indicates that *B. bassiana* coevolved beetles are neither positively or negatively cross-resistant to *P. entomophila* upon systemic infection.

In the *B. bassiana* assay, survival of groups in INFECTION and CONTROL treatments were significantly different from each other (χ^2^ = 41.1, DF = 3, *p* < 0.001). Pairwise comparison of Kaplan-Meier curves (Figure 1c and Table 1) revealed that ‘Control’ and ‘Coevolved’ groups differ from each other within CONTROL (*p* = 0.004), but not within INFECTION treatment (*p* = 0.397), with the ‘Control’ group surviving better than ‘Coevolved’ group.

### 3.2. Gene Expression Analysis Reveals Expression of Markers for Oral Toxicity upon B. bassiana Exposure

**Figure 2:**
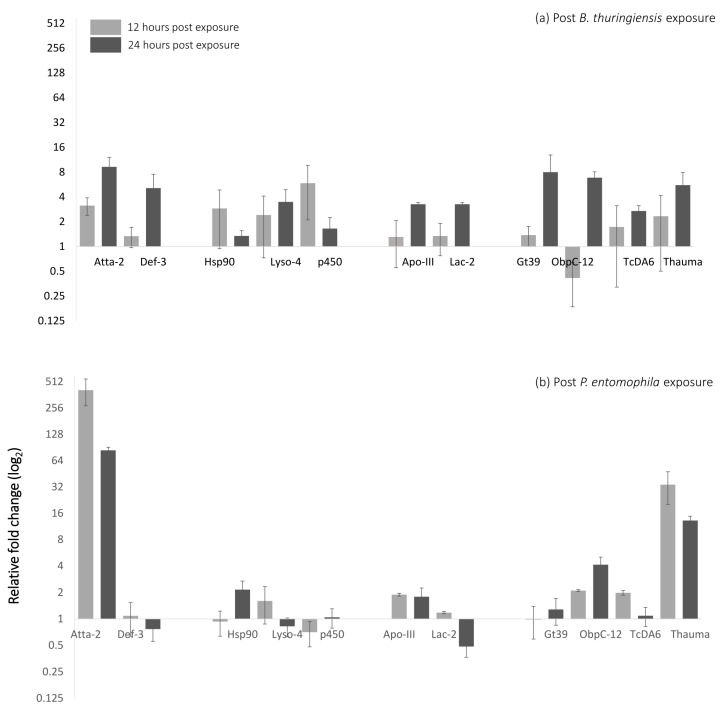

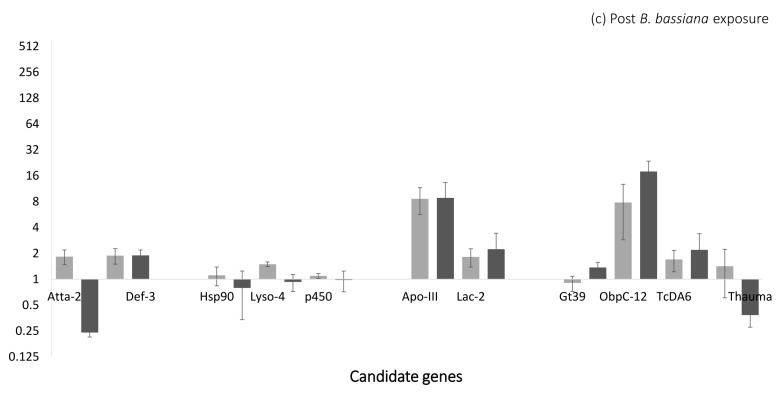
RT-qPCR results on the coevolved beetles (relative to coevolved beetles not exposed to parasites) upon exposure with ((**a**) *B.* *thuringiensis*, (**b**) *P. entomophila* and (**c**) non-coevolved *B. bassiana*, 12 and 24 h post exposure (see Supplementary Materials File 5, Table S1 for fold change values). The route of parasite entry for the qPCR was kept the same as that used for the survival assay. (Error bars indicate (±) standard errors of the mean).

We used RT-qPCR to investigate differences in the expression of 11 candidate genes post *B. bassiana, B. thuringiensis* and *P. entomophila* on F2 larvae of generation 12 beetles evolved with *B. bassiana* under conditions allowing for coevolution. The larvae were sampled for RT-qPCR experiments 12 and 24 h post exposure. Most of the candidate genes were up-regulated 12 h post *B. thuringiensis* exposure with markers for stress, Hsp90 (3 fold) and p450 (6.6 fold) being prominent (Figure 2a), implying cross-talk between immune and stress response [39,49]. 24 h post *B. thuringiensis* exposure showed up-regulation in all of the candidate genes. When compared to 12 h, stress markers Hsp90 (1.35 fold) and p450 (1.65 fold) were less up-regulated. Additionally, the innate immunity marker, Lysozyme was up-regulated (Figure 2a). 12 h post *P. entomophila* exposure Attacin-2 (411 fold) and Thaumatin (34 fold) were highly up-regulated (Figure 2b). This observation is in consensus with previous findings reporting the expression of these genes upon Gram negative bacterial exposure [49,50,51]. Here, no change in gene expression pattern (up or down-regulation) was observed at 24 h compared to 12 h, expression levels of Attacin-2 (84 fold) and Thaumatin (13.22 fold) decreased (Figure 2b). 12 h post *B. bassiana* exposure, Apo-III (8.63 fold) and ObpC-12 (~8 fold) were up-regulated but not Thaumatin (Figure 2c). Upregulation of Apo-III (8.84 fold) and ObpC-12 (18 fold) was observed 24 post *B. bassiana* exposure as well (Figure 2c).

**Figure 3:**
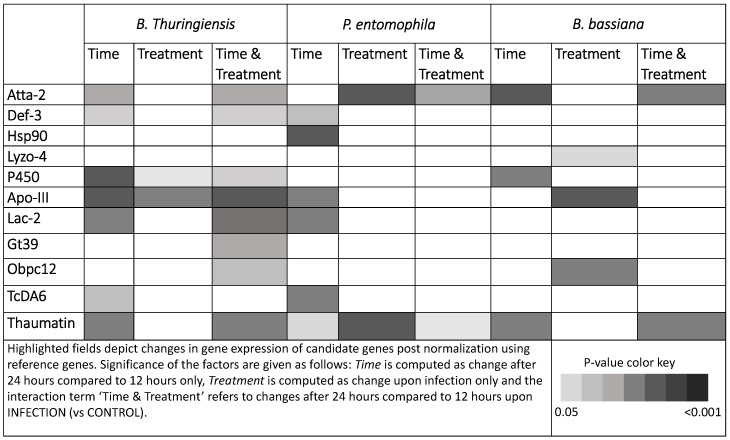
Results from the analysis of differential relative gene expression upon parasite exposure via generalized linear mixed models performed using the R package MCMC.qpcr [47,48]. See Supplementary Materials File 5, Table S2 for corresponding *p*-values.

Changes in gene expression upon *B. thuringiensis*, *P. entomophila* and *B. bassiana* exposures with respect to time (12 vs. 24 h) and treatment (CONTROL vs. INFECTION) are summarized in Figure 3. Here too, differential expression of greater number of candidate upon B. thuringiensis infection than other exposures (Figure 3) is observed. In general, we observed more genes being differentially expressed with respect to time than to treatment.

## 4. Discussion

Here, we present the first report of evolutionary positive cross-resistance in *T. castaneum,* as a consequence of coevolution with *B. bassiana,* which is shown towards *B. thuringiensis* upon oral exposure, but not upon systemic exposure with *P. entomophila*. This observation is mirrored in expression pattern of host genes related to resistance to oral toxicity upon *B. bassiana* exposure. It has been reported that oral infection with *B. thuringiensis* and exposure to coleopteran specific *Cry*-III toxins leads to the expression of several odorant-binding proteins [30,52] and Apolipophorins [30,42] in *T. castaneum*. We observed an upregulation in Apo-III and ObpC-12, our markers for oral infection, upon *B. bassiana* exposure. Infection with Gram-negative *P. entomophila* caused no variation in coevolved *T. castaneum* larvae neither in survival assay nor qPCR. The up-regulation of Atta-2 is consistent with reports that show the expression of this anti-microbial peptide upon Gram-negative bacteria infection [51]. Due to the constant presence of *B. bassiana* during coevolution [32], there was a high chance of *T. castaneum* ingesting *B. bassiana* spores. Furthermore, there is some evidence that *B. bassiana* may be able to infect orally [53,54], although this is yet to be mechanistically proven. This occurrence potentially led to *T. castaneum* adapting to oral infection. This is consistent with previous findings that the coevolved beetles have more flexible PO responses that vary depending on infection route, indicating that during the course of coevolution, *B. Bassiana* adapted to infecting the host orally [33]. Together, the response of coevolved beetles to *B. bassiana* and *B. thuringiensis* exposure imply that similar defence mechanisms are effective against both of the parasites. Here, it is imperative to mention that the overserved response of the coevolved hosts could have been influenced by carried over maternal effects, due to constant exposure of the previous generations to parasites in the environment. However, immune priming has not been consistently observed in insects exposed to *B. bassiana* [55] and to date all transgenerational immune priming seen in *T. castaneum* has been over a single generation [36,37,56]. Therefore, parental priming to *B. bassiana* is unlikely to be an adequate explanation to our results.

Positive cross-resistance is more likely to occur if the defence mechanisms employed by the host towards the two parasites are similar or shared, owing to a similarity in the route of parasite entry and/or mechanism of infection [23]. Oral toxicity of *B. thuringiensis* is mediated by the production of *Cry* toxins that solubilise in the insect midgut due to a change in pH and disrupt peritrophic membrane integrity [57,58]. *B. bassiana* is known to infect by germination of spores on the insect cuticle followed by cuticular breaching through hyphae [53,59]. That *B. bassiana* possesses the ability to infect orally has been a matter of speculation with some experimental evidence [54,60]. For example, in the red imported ant *Solenopsis invicta, B. bassiana* was shown to successfully infect via oral ingestion of conidia [54]. Furthermore, through comparative genome analysis, Xiao and colleagues found that in contrast to other entomopathogenic fungi, *B. bassiana* possess *Cry*-like toxin coding regions [61]. Our findings of cross-resistance towards *B. thuringiensis* upon coevolution with *B. bassiana* coupled with the expression of oral infection marker upon *B. bassiana* exposure, suggest that *B. bassiana* and *B. thuringiensis* share a similar infection route.

Behrens et al. [30] showed that the transcriptomic response of *T. castaneum* larvae differs based on the natural (oral) and artificial (systemic) routes via which *B. thuringiensis* infects the beetle. Experimental evolution of *D. melanogaster* to *P. entomophila* via oral and systemic routes, separately, revealed that adaptation to different routes was specific; flies adapted to one infection route were not resistant to *P. entomophila* infection via the route that they had not evolved to [10]. Indeed, the route of infection is important as the host physiological response may vary based on different routes. Our observations that the candidate gene for oral infection (ObpC-12) is expressed upon *B. bassiana* infection coupled with the fact that genomic analyses of *B. bassiana* reveals potential oral toxicity [61] and that *B. bassiana* coevolved beetles are positively cross-resistant to *B. thuringiensis* supports the argument that *B. bassiana* is able to infect orally [54,61].

Experimental evolution of insect hosts with *B. bassiana* provides contrasting results in terms of evolution of host resistance. While increase in resistance to *B. bassiana* was reported for *G. melonella* [11], evolved populations of *D. melanogaster* displayed no change in resistance towards *B. bassiana* [62]. In our study, ‘Coevolved’ *T. castaneum* larvae showed no difference in survival following exposure to *B. bassiana* when compared to ‘Control’ larvae. They do, however, buffer their fitness across evolutionary time by maintaining, or in the highest infection load treatment increasing pupae numbers when challenged with *B. bassiana* during coevolution and show infection route specific changes in phenoloxidase activity [33], implying some level of underlying resistance towards *B. bassiana*. The increased survival we observe here following *B. thuringiensis* infection adds support to the hypothesis that oral infection by *B. bassiana* drove selection specifically against oral infection.

Our results also have wider ecological and applied implications. *B. bassiana* spore suspensions and *B. thuringiensis* strains specific to different pest insect orders are widely used as biological control agents [63,64]. Prior exposure to *B. bassiana* could potentially lead to positive ecological cross-resistance in pest insects when exposed to *B. thuringiensis* strains and vice-versa, with implications in sustainable pest management. Positive cross-resistance can be beneficially applied by providing controlled doses of a parasite that protects against attacks by a more harmful parasite. This is similar to the application of probiotics in the culture of insects for food and feed [65]. Further research is warranted in the beneficial effects of positive cross-resistance for rearing beneficial insects.

## 5. Conclusions

We observe positive cross resistance of *T. castaneum* beetles coevolved with *B. bassiana* towards *B. thuringiensis*. We propose that this observation could be based on similarity in the route of entry and/or mechanism of infection between the two parasites. Supporting this hypothesis, RT-qPCR experiments performed in this study indicate that *B. bassiana* can induce expression of host genes that are related to oral toxicity. Adaptations of beetles to oral infection by *B. bassiana* may have led to positive cross-resistance in the coevolved beetles upon infection with *B. thuringiensis*. We thereby support the fact that the route of infection is highly important in host-parasite interactions and the physiological response of the host, as well as that of the parasite warrant more research.

## Figures and Tables

**Table 1:** Results (*p*-values) of pairwise-wise comparison of survival curves using log-rank tests followed by Holm correction.

Origin × Treatment	*B. thuringiensis* Survival Assay		
	‘Coevolved’ CONTROL	‘Control’ CONTROL	‘Coevolved’ INFECTION
‘Control’ CONTROL	0.05		
‘Coevolved’ INFECTION	<0.001	<0.001	
‘Control’ INFECTION	<0.001	<0.001	<0.001
	*P. entomophila* survival assay		
‘Control’ CONTROL	0.19		
‘Coevolved’ INFECTION	<0.001	<0.001	
‘Control’ INFECTION	<0.001	<0.001	0.80
	*B. bassiana* survival assay		
‘Control’ CONTROL	0.004		
‘Coevolved’ INFECTION	0.003	<0.001	
‘Control’ INFECTION	0.022	<0.001	0.397

Note: ‘Control’ and ‘Coevolved’ denote the evolutionary background of the hosts; CONTROL and INFECTION refer to the treatments in the survival assay.

## Supplementary Materials

The following are available online at http://www.mdpi.com/2075-4450/9/1/28/s1, File 1: Survival data, File 2: Information regarding the primers used for RT-qPCR, File 3: qPCR 12 h, File 4: q PCR 24 h, File 5: Results from gene expression.

## References

[1] Bargielowski I., Koella J.C. (2009). A Possible Mechanism for the Suppression of *Plasmodium berghei* Development in the Mosquito *Anopheles gambiae* by the Microsporidian *Vavraia culicis*. PLoS ONE.

[2] Betts A., Rafaluk C., King K.C. (2016). Host and Parasite Evolution in a Tangled Bank. Trends Parasitol..

[3] Hafer N., Milinski M. (2016). Inter- and intraspecific conflicts between parasites over host manipulation. Proc. R. Soc. B.

[4] Bordes F., Morand S. (2009). Coevolution between multiple helminth infestations and basal immune investment in mammals: Cumulative effects of polyparasitism?. Parasitol. Res..

[5] Start D., Gilbert B. (2016). Host–parasitoid evolution in a metacommunity. Proc. R. Soc. B.

[6] De Roode J.C., Culleton R., Cheesman S.J., Carter R., Read A.F. (2004). Host heterogeneity is a determinant of competitive exclusion or coexistence in genetically diverse malaria infections. Proc. Biol. Sci..

[7] Hatcher M.J., Dick J.T., Dunn A.M. (2012). Diverse effects of parasites in ecosystems: Linking interdependent processes. Front. Ecol. Environ..

[8] Von Beeren C., Maruyama M., Hashim R., Witte V. (2010). Differential host defense against multiple parasites in ants. Evol. Ecol..

[9] Fellowes M.D.E., Kraaijeveld A.R., Godfray H.C.J. (1999). Cross-Resistance Following Artificial Selection for Increased Defense against Parasitoids in *Drosophila melanogaster*. Evolution.

[10] Martins N.E., Faria V.G., Teixeira L., Magalhães S., Sucena É. (2013). Host Adaptation Is Contingent upon the Infection Route Taken by Pathogens. PLoS Pathog..

[11] Dubovskiy I.M., Whitten M.M.A., Yaroslavtseva O.N., Greig C., Kryukov V.Y., Grizanova E.V., Mukherjee K., Vilcinskas A., Glupov V.V., Butt T.M. (2013). Can Insects Develop Resistance to Insect Pathogenic Fungi?. PLoS ONE.

[12] Faria V.G., Martins N.E., Paulo T., Teixeira L., Sucena É., Magalhães S. (2015). Evolution of *Drosophila* resistance against different pathogens and infection routes entails no detectable maintenance costs. Evolution.

[13] Martins N.E., Faria V.G., Nolte V., Schlötterer C., Teixeira L., Sucena É., Magalhães S. (2014). Host adaptation to viruses relies on few genes with different cross-resistance properties. Proc. Natl. Acad. Sci. USA.

[14] Decaestecker E., Gaba S., Raeymaekers J.A.M., Stoks R., Van Kerckhoven L., Ebert D., De Meester L. (2007). Host–parasite ‘Red Queen’ dynamics archived in pond sediment. Nature.

[15] Schulte R.D., Makus C., Hasert B., Michiels N.K., Schulenburg H. (2011). Host–parasite local adaptation after experimental coevolution of *Caenorhabditis elegans* and its microparasite *Bacillus thuringiensis*. Proc. R. Soc. Lond. B Biol. Sci..

[16] Schulte R.D., Makus C., Hasert B., Michiels N.K., Schulenburg H. (2010). Multiple reciprocal adaptations and rapid genetic change upon experimental coevolution of an animal host and its microbial parasite. Proc. Natl. Acad. Sci. USA.

[17] Bérénos C., Schmid-Hempel P., Wegner K.M. (2012). Complex adaptive responses during antagonistic coevolution between Tribolium castaneum and its natural parasite *Nosema whitei* revealed by multiple fitness components. BMC Evol. Biol..

[18] Bérénos C., Schmid-Hempel P., Mathias Wegner K. (2009). Evolution of host resistance and trade-offs between virulence and transmission potential in an obligately killing parasite. J. Evol. Biol..

[19] Bérénos C., Wegner K.M., Schmid-Hempel P. (2010). Antagonistic coevolution with parasites maintains host genetic diversity: An experimental test. Proc. R. Soc. Lond. B Biol. Sci..

[20] Dupas S., Carton Y., Poiriè M. (2003). Genetic dimension of the coevolution of virulence–resistance in *Drosophila*––Parasitoid wasp relationships. Heredity.

[21] Kerstes N.A., Bérénos C., Schmid-Hempel P., Wegner K.M. (2012). Antagonistic experimental coevolution with a parasite increases host recombination frequency. BMC Evol. Biol..

[22] Kraaijeveld A.R., Godfray H.C.J. (1997). Trade-off between parasitoid resistance and larval competitive ability in *Drosophila melanogaster*. Nature.

[23] Kraaijeveld A.R., Layen S.J., Futerman P.H., Godfray H.C.J. (2012). Lack of Phenotypic and Evolutionary Cross-Resistance against Parasitoids and Pathogens in *Drosophila melanogaster*. PLoS ONE.

[24] Zuk M., Stoehr A.M. (2002). Immune Defense and Host Life History. Am. Nat..

[25] Koskella B., Lin D.M., Buckling A., Thompson J.N. (2012). The costs of evolving resistance in heterogeneous parasite environments. Proc. R. Soc. Lond. B Biol. Sci..

[26] Scarborough C.L., Ferrari J., Godfray H.C.J. (2005). Aphid Protected from Pathogen by Endosymbiont. Science.

[27] Parker B.J., Spragg C.J., Altincicek B., Gerardo N.M. (2013). Symbiont-Mediated Protection against Fungal Pathogens in Pea Aphids: A Role for Pathogen Specificity?. Appl. Environ. Microbiol..

[28] Xie J., Butler S., Sanchez G., Mateos M. (2014). Male killing *Spiroplasma* protects *Drosophila melanogaster* against two parasitoid wasps. Heredity.

[29] Bentz M.L., Humphrey E.A., Harshman L.G., Wayne M.L. (2017). Sigma Virus (DMelSV) Incidence in Lines of *Drosophila melanogaster* Selected for Survival following Infection with *Bacillus cereus*. Psyche J. Entomol..

[30] Behrens S., Peuß R., Milutinović B., Eggert H., Esser D., Rosenstiel P., Schulenburg H., Bornberg-Bauer E., Kurtz J. (2014). Infection routes matter in population-specific responses of the red flour beetle to the entomopathogen *Bacillus thuringiensis*. BMC Genomics.

[31] Brockhurst M.A., Koskella B. (2013). Experimental coevolution of species interactions. Trends Ecol. Evol..

[32] Rafaluk C., Yang W., Mitschke A., Rosenstiel P., Schulenburg H., Joop G. (2017). Highly potent host external immunity acts as a strong selective force enhancing rapid parasite virulence evolution. Environ. Microbiol..

[33] Rafaluk-Mohr C., Wagner S., Joop G. (2018). Cryptic changes in immune response and fitness in *Tribolium castaneum* as a consequence of coevolution with *Beauveria bassiana*. J. Invertebr. Pathol..

[34] Vodovar N., Vallenet D., Cruveiller S., Rouy Z., Barbe V., Acosta C., Cattolico L., Jubin C., Lajus A., Segurens B. (2006). Complete genome sequence of the entomopathogenic and metabolically versatile soil bacterium *Pseudomonas entomophila*. Nat. Biotechnol..

[35] Milutinović B., Stolpe C., Peuβ R., Armitage S.A.O., Kurtz J. (2013). The Red Flour Beetle as a Model for Bacterial Oral Infections. PLoS ONE.

[36] Roth O., Joop G., Eggert H., Hilbert J., Daniel J., Schmid-Hempel P., Kurtz J. (2010). Paternally derived immune priming for offspring in the red flour beetle, *Tribolium castaneum*. J. Anim. Ecol..

[37] Roth O., Sadd B.M., Schmid-Hempel P., Kurtz J. (2009). Strain-specific priming of resistance in the red flour beetle, *Tribolium castaneum*. Proc. R. Soc. Lond. B Biol. Sci..

[38] Bustin S.A., Benes V., Garson J.A., Hellemans J., Huggett J., Kubista M., Mueller R., Nolan T., Pfaffl M.W., Shipley G.L. (2009). The MIQE Guidelines: Minimum Information for Publication of Quantitative Real-Time PCR Experiments. Clin. Chem..

[39] Altincicek B., Knorr E., Vilcinskas A. (2008). Beetle immunity: Identification of immune-inducible genes from the model insect *Tribolium castaneum*. Dev. Comp. Immunol..

[40] Arakane Y., Muthukrishnan S., Beeman R.W., Kanost M.R., Kramer K.J. (2005). Laccase 2 is the phenoloxidase gene required for beetle cuticle tanning. Proc. Natl. Acad. Sci. USA.

[41] Arakane Y., Dixit R., Begum K., Park Y., Specht C.A., Merzendorfer H., Kramer K.J., Muthukrishnan S., Beeman R.W. (2009). Analysis of functions of the chitin deacetylase gene family in *Tribolium castaneum*. Insect Biochem. Mol. Biol..

[42] Contreras E., Rausell C., Real M.D. (2013). *Tribolium castaneum* Apolipophorin-III acts as an immune response protein against *Bacillus thuringiensis* Cry3Ba toxic activity. J. Invertebr. Pathol..

[43] Li J., Lehmann S., Weißbecker B., Ojeda Naharros I., Schütz S., Joop G., Wimmer E.A. (2013). Odoriferous Defensive stink gland transcriptome to identify novel genes necessary for quinone synthesis in the red flour beetle, *Tribolium castaneum*. PLoS Genet..

[44] Lord J.C., Hartzer K., Toutges M., Oppert B. (2010). Evaluation of quantitative PCR reference genes for gene expression studies in *Tribolium castaneum* after fungal challenge. J. Microbiol. Methods.

[45] R Development Core Team (2008). R: A Language and Environment for Statistical Computing.

[46] Therneau T. A Package for Survival Analysis in S. R Package Version 2.37-4. https://cran.r-project.org/web/packages/survival/index.html.

[47] Matz M.V., Wright R.M., Scott J.G. (2013). No Control Genes Required: Bayesian Analysis of qRT-PCR Data. PLoS ONE.

[48] Matz M.V. (2016). MCMC.qpcr: Bayesian Analysis of qRT-PCR Data. https://cran.r-project.org/web/packages/MCMC.qpcr/index.html.

[49] Altincicek B., Elashry A., Guz N., Grundler F.M.W., Vilcinskas A., Dehne H.-W. (2013). Next Generation Sequencing Based Transcriptome Analysis of Septic-Injury Responsive Genes in the Beetle *Tribolium castaneum*. PLoS ONE.

[50] Kim Y.-S., Ryu J.-H., Han S.-J., Choi K.-H., Nam K.-B., Jang I.-H., Lemaitre B., Brey P.T., Lee W.-J. (2000). Gram-negative Bacteria-binding Protein, a Pattern Recognition Receptor for Lipopolysaccharide and β-1,3-Glucan That Mediates the Signaling for the Induction of Innate Immune Genes in *Drosophila melanogaster* Cells. J. Biol. Chem..

[51] Gottar M., Gobert V., Michel T., Belvin M., Duyk G., Hoffmann J.A., Ferrandon D., Royet J. (2002). The *Drosophila* immune response against Gram-negative bacteria is mediated by a peptidoglycan recognition protein. Nature.

[52] Contreras E., Rausell C., Real M.D. (2013). Proteome Response of *Tribolium castaneum* Larvae to *Bacillus thuringiensis* Toxin Producing Strains. PLoS ONE.

[53] Pekrul S., Grula E.A. (1979). Mode of infection of the corn earworm (Heliothis zea) by *Beauveria bassiana* as revealed by scanning electron microscopy. J. Invertebr. Pathol..

[54] Siebeneicher S.R., BradleighˆVinson S., Kenerley C.M. (1992). Infection of the red imported fire ant by *Beauveria bassiana* through various routes of exposure. J. Invertebr. Pathol..

[55] Reber A., Chapuisat M. (2012). No Evidence for Immune Priming in Ants Exposed to a Fungal Pathogen. PLoS ONE.

[56] Knorr E., Schmidtberg H., Arslan D., Bingsohn L., Vilcinskas A. (2015). Translocation of bacteria from the gut to the eggs triggers maternal transgenerational immune priming in *Tribolium castaneum*. Biol. Lett..

[57] Bravo A., Gill S.S., Soberón M. (2007). Mode of action of *Bacillus thuringiensis* Cry and Cyt toxins and their potential for insect control. Toxicon.

[58] Pardo-López L., Soberón M., Bravo A. (2013). *Bacillus thuringiensis* insecticidal three-domain Cry toxins: mode of action, insect resistance and consequences for crop protection. FEMS Microbiol. Rev..

[59] Rice W.C., Cogburn R.R. (1999). Activity of the Entomopathogenic Fungus *Beauveria bassiana* (Deuteromycota: Hyphomycetes) Against Three Coleopteran Pests of Stored Grain. J. Econ. Entomol..

[60] Yanagita T. (1987). Studies on oral infection of larvae of the silkworm, *Bombyx mori*, with *Beauveria bassiana*. J. Sericultural Sci. Jpn..

[61] Xiao G., Ying S.-H., Zheng P., Wang Z.-L., Zhang S., Xie X.-Q., Shang Y., St. Leger R.J., Zhao G.-P., Wang C. (2012). Genomic perspectives on the evolution of fungal entomopathogenicity in *Beauveria bassiana*. Sci. Rep..

[62] Kraaijeveld A.R., Godfray H.C.J. (2008). Selection for resistance to a fungal pathogen in *Drosophila melanogaster*. Heredity.

[63] Lacey L.A., Frutos R., Kaya H.K., Vail P. (2001). Insect Phiathogens as Biological Control Agents: Do They Have a Future?. Biol. Control.

[64] Lacey L.A., Grzywacz D., Shapiro-Ilan D.I., Frutos R., Brownbridge M., Goettel M.S. (2015). Insect pathogens as biological control agents: Back to the future. J. Invertebr. Pathol..

[65] Grau T., Vilcinskas A., Joop G. (2017). Sustainable farming of the mealworm *Tenebrio molitor* for the production of food and feed. Z. Naturforschung C.

