# Supplementary material for: Cross-Resistance: A Consequence of Bi-partite Host-Parasite Coevolution
Source: Insects. 2018 Feb 26;9(1):28. doi: 10.3390/insects9010028 (PMC5872293; doi:10.3390/insects9010028)
Supplement: Supplementary file 1 [file insects-09-00028-s001.zip › Supplementary material/Supplementary Materials File 2-information regarding primers.docx]

**Supplementary Materials File 2. Information regarding the primers used for RT-qPCR.**

**Table S1.** List of primers for candidate genes along with their efficiencies used for RT-qPCR.

| **Gene** | **Function** | **Gene ID (sourced from NCBI)** | **forward primer** | **reverse primer** | **EFFICEINCY (85-110%)** | **R^2 (>=0.98)** |
| --- | --- | --- | --- | --- | --- | --- |
| **Rps3**[1] ***** | *T. castaneum* ribosomal protein S3 (RpS3), mRNA. (reference gene) | NM_001172392.1 | ACCTCGATACACCATAGCAAGC | ACCGTCGTATTCGTGAATTGAC | 96.67 | 0.994 |
| **Rps18** [1] | Ribosomal protein (reference gene) | XM_968539.2 | TGATGGCAAACGCAAAGTCA | TCGGCCGACACCTTTGA | 93.809 | 0.985 |
| **Attacin-2 (Atta-2)** | anti-microbial peptide | 100141947 | AGTCGGCGTTGAAGCATC | CCCGAACCTCTGACCATAG | 89.595 | 0.995 |
| **Defensin 3** | Anti-microbial peptide mainly active against Gram negative bacteria | 655548 | TGTCACACTAGTGATGGGGC | ATCATTCTTTTGGTGTCCCG | 85.154 | 0.997 |
| **Hsp90** | Heat shock protein; *marker for systemic infection* | 656270 | CCTCAAGTCCACGCATCCAG | TCGCCTCCTTGTGCATCTTC | 110.7 | 0.989 |
| **Lysozyme4 (Lyzo-4)** | General immune molecule | NM_00116**6**023.1 | TGAGTTAGCCCGCGAATTGAA | TAGCCATTGCCAGGTGGTGA | 85.419 | 0.997 |
| **p450** | Oxidative stress; *marker for systemic infection* | 470011965 | GGGGTTTGGTCACAGATGATG | CATTGCCGTGGATGATGTCTC | 91.721 | 0.982 |
| **Apolipophorin-III (Apo-III)** [2] | Activated upon coleopteran specific *Cry* toxin challenge (proPO Candidate); *marker for oral infection* | 655732 | CCAAAACGCCGCTCAAAC | TTGCAAATTGTTGCTGACTTCA | 107.682 | 0.993 |
| **Laccase2 (Lac-2)** | PO candidate that is responsible for cuticular darkening | 641461 | TCTGCGAAGGTGACAAGGTTG | GGGGCATTGGGTAACGAAAG | 92.856 | 0.98 |
| **Tcas-ql VTGI (Gt39)** [3] | Quinone related; external immune defense | JX569829.1 | TACACTTCTCCACCTGACAATG | ACCCATACCTGGTTTTCGTAC | 95.139 | 0.992 |
| **ObpC-12** | Odorant binding protein; *marker for oral infection* | 656243 | CAAGCCAAACAGCTAAGGAA | CCCGACACCGACTTGCA | 95.622 | 0.992 |
| **TcCDA6** [4] | Chitin deacetylase active in larval gut | NM_0011**1**0435**.**1 | CGGCAGAGTACTGGTTGAAAGC | CAATGGGAATGTTGGCAAAGTG | 85.959 | 0.983 |
| **Thaumatin-like (Thaumatin)** [5] | Expressed upon fungal challenge | 663483 | GGCAACGGGGTTATTGCTTG | ACGTGTCAGGTGTGCCGAAA | 97.86 | 0.982 |

* Citations here refer to the publications from which the respective primer sequences have been adopted.

**Table S2.** Primer concentrations and melting temperatures.

| **gene** | **Forward primer T_m_ (°C)** | **Reverse primer T_m_ (°C)** | **Forward primer concentration (nM)** | **Reverse primer concentration (nM)** |
| --- | --- | --- | --- | --- |
| hsp90 | 60.74 | 60.39 | 300 | 150 |
| Thaumatin | 60.11 | 62.58 | 500 | 500 |
| ApoIII | 58.08 | 57.88 | 900 | 300 |
| Rps3 | 60.22 | 59.08 | 300 | 150 |
| Obp-C12 | 56.24 | 59.60 | 300 | 300 |
| Rps18 | 58.97 | 58.42 | 500 | 300 |
| Lac2 | 57.63 | 57.81 | 300 | 150 |
| Lyzo | 60.34 | 61.50 | 500 | 300 |
| TcDA6 | 65.5 | 66.7 | 300 | 300 |
| p450 | 64.8 | 66.1 | 300 | 300 |
| atta2 | 63.3 | 64.2 | 150 | 150 |
| gt39 | 60 | 60 | 500 | 500 |
| def3 | 59.39 | 56.02 | 300 | 500 |

T_m_ = melting temperature.

**References**

1. Lord, J.C.; Hartzer, K.; Toutges, M.; Oppert, B. Evaluation of quantitative PCR reference genes for gene expression studies in Tribolium castaneum after fungal challenge. *J. Microbiol. Methods* **2010**, *80*, 219–221, doi:10.1016/j.mimet.2009.12.007.

2. Contreras, E.; Rausell, C.; Real, M.D. Tribolium castaneum Apolipophorin-III acts as an immune response protein against Bacillus thuringiensis Cry3Ba toxic activity. *J. Invertebr. Pathol.* **2013**, *113*, 209–213, doi:10.1016/j.jip.2013.04.002.

3. Li, J.; Lehmann, S.; Weißbecker, B.; Ojeda Naharros, I.; Schütz, S.; Joop, G.; Wimmer, E.A. Odoriferous Defensive stink gland transcriptome to identify novel genes necessary for quinone synthesis in the red flour beetle, Tribolium castaneum. *PLoS Genet.* **2013**, *9*, e1003596, doi:10.1371/journal.pgen.1003596.

4. Arakane, Y.; Dixit, R.; Begum, K.; Park, Y.; Specht, C.A.; Merzendorfer, H.; Kramer, K.J.; Muthukrishnan, S.; Beeman, R.W. Analysis of functions of the chitin deacetylase gene family in Tribolium castaneum. *Insect Biochem. Mol. Biol.* **2009**, *39*, 355–365, doi:10.1016/j.ibmb.2009.02.002.

5. Altincicek, B.; Knorr, E.; Vilcinskas, A. Beetle immunity: Identification of immune-inducible genes from the model insect Tribolium castaneum. *Dev. Comp. Immunol.* **2008**, *32*, 585–595, doi:10.1016/j.dci.2007.09.005.

© 2018 by the authors. Submitted for possible open access publication under the terms and conditions of the Creative Commons Attribution (CC BY) license (http://creativecommons.org/licenses/by/4.0/).
