# Supplementary material for: Cross-Resistance: A Consequence of Bi-partite Host-Parasite Coevolution
Source: Insects. 2018 Feb 26;9(1):28. doi: 10.3390/insects9010028 (PMC5872293; doi:10.3390/insects9010028)
Supplement: Supplementary file 1 [file insects-09-00028-s001.zip › Supplementary material/Supplementary Materials File 5-qPCR results.docx]

**Supplementary Materials File 5. Results from gene expression.**

**Table S1.** Fold change values from qPCR reactions, relative to CONTROL treatment. Downward arrows indicate down-regulation of genes.

|  | *B. thuringiensis* | | *P. entomphila* | | *B. bassiana* | |
| --- | --- | --- | --- | --- | --- | --- |
|  | 12 hours | 24 hours | 12 hours | 24 hours | 12 hours | 24 hours |
| **Hsp90** | 2.903 | 1.348 | 1.074 **↓** | 2.152 | 1.112 | 1.264 **↓** |
| **Lysosyme** | 2.419 | 3.486 | 1.600 | 1.212 **↓** | 1.494 | 1.076 **↓** |
| **P450** | 5.882 | 1.652 | 1.414 ↓ | 1.044 | 1.094 | 1.024 **↓** |
| **Attacin 2** | 3.147 | 9.282 | 410.791 | 83.972 | 1.832 | 4.270 **↓** |
| **Defensin 3** | 1.340 | 5.127 | 1.083 | 1.305 **↓** | 1.882 | 1.893 |
| **Laccase 2** | 1.343 | 3.262 | 1.797 | 2.061 **↓** | 1.822 | 2.230 |
| **TcDA6 (chitin deacetylase)** | 1.730 | 2.705 | 1.980 | 1.088 | 1.694 | 2.198 |
| **ApoIII** | 1.307 | 3.262 | 1.886 | 1.786 | 8.631 | 8.840 |
| **Obpc12** | 2.40 **↓** | 6.858 | 2.101 | 4.137 | 7.796 | 17.902 |
| **GT39 (quinone)** | 1.376 | 8.00 | 1.219 | 1.280 | 1.108 | 1.369 |
| **Thaumatin** | 2.339 | 5.564 | 33.993 | 12.219 | 1.418 | 2.617 **↓** |

**Table S2.** P-values of MCMC analysis of qPCR fold change data. The up and down arrows indicate relative expression pattern.

|  | ***B. thuringiensis*** | | | ***P. entomophila*** | | | ***B. bassiana*** | | |
| --- | --- | --- | --- | --- | --- | --- | --- | --- | --- |
|  | Time  (12 vs 24 hours) | Treatment  (CONTROL vs INFECTION) | Time & Treatment | Time  (12 vs 24 hours) | Treatment (CONTROL vs INFECTION) | Time & Treatment | Time  (12 vs 24 hours) | Treatment (CONTROL vs INFECTION) | Time & Treatment |
| **Hsp90** |  |  |  | <0.001 **↑** |  |  |  |  |  |
| **Lysosyme** | <0.001 **↓** | 0.046 **↓** | 0.036 **↓** |  |  |  |  |  |  |
| **P450** | 0.014 **↓** |  | 0.024 **↓** |  |  |  | <0.001 **↓** | 0.046 **↓** | 0.036 **↓** |
| **Attacin 2** | 0.020 **↑** |  | 0.014 **↑** |  | <0.001 **↑** | 0.010 **↑** | 0.014 **↓** |  | 0.024 **↓** |
| **Defensin 3** | <0.001 **↑** |  | 0.002 **↑** | 0.012 **↑** |  |  | 0.020 **↑** |  | 0.014 **↑** |
| **Laccase 2** | 0.022 **↓** |  |  | 0.002 **↑** |  |  | <0.001 **↑** |  | 0.002 **↑** |
| **TcDA6 (chitin deacetylase)** | <0.001 **↑** | 0.002 **↑** | <0.001 **↑** | 0.002 **↑** |  |  | 0.022 **↓** |  |  |
| **ApoIII** |  |  | 0.016 **↑** | 0.004 **↑** |  |  | <0.001 **↑** | 0.002 **↑** | <0.001 **↑** |
| **Obpc12** |  |  | 0.012 **↓** |  |  |  |  |  | 0.016 **↑** |
| **GT39 (quinone)** | 0.004 **↓** |  | 0.002 **↓** |  |  |  |  |  | 0.012 **↓** |
| **Thaumatin** | 0.002 **↓** |  | 0.004 **↓** | 0.040 **↑** | <0.001 **↑** | 0.046 **↑** | 0.004 **↓** |  | 0.002 **↓** |

© 2018 by the authors. Submitted for possible open access publication under the terms and conditions of the Creative Commons Attribution (CC BY) license (http://creativecommons.org/licenses/by/4.0/).
